# Radiological, clinical, and molecular analyses reveal distinct subtypes of butterfly glioblastomas affecting the prognosis

**DOI:** 10.1093/noajnl/vdae180

**Published:** 2024-10-23

**Authors:** Ichiyo Shibahara, Ryota Shigeeda, Takashi Watanabe, Yasushi Orihashi, Yoko Tanihata, Kazuko Fujitani, Hajime Handa, Yuri Hyakutake, Mariko Toyoda, Madoka Inukai, Kohei Uemasu, Mitsuhiro Shinoda, Hideto Komai, Sumito Sato, Takuichiro Hide, Toshihiro Kumabe

**Affiliations:** Department of Neurosurgery, Kitasato University School of Medicine, Sagamihara, Kanagawa, Japan; Department of Neurosurgery, Kitasato University School of Medicine, Sagamihara, Kanagawa, Japan; Department of General Internal Medicine, JCHO Sendai Hospital, Sendai, Miyagi, Japan; Clinical Research Center in Hiroshima, Hiroshima University Hospital, Hiroshima, Hiroshima, Japan; Department of Neurosurgery, Kitasato University School of Medicine, Sagamihara, Kanagawa, Japan; Gene Analysis Center, Kitasato University School of Medicine, Sagamihara, Kanagawa, Japan; Department of Neurosurgery, Kitasato University School of Medicine, Sagamihara, Kanagawa, Japan; Department of Neurosurgery, Kitasato University School of Medicine, Sagamihara, Kanagawa, Japan; Department of Neurosurgery, Kitasato University School of Medicine, Sagamihara, Kanagawa, Japan; Department of Neurosurgery, Kitasato University School of Medicine, Sagamihara, Kanagawa, Japan; Department of Neurosurgery, Kitasato University School of Medicine, Sagamihara, Kanagawa, Japan; Department of Neurosurgery, Kitasato University School of Medicine, Sagamihara, Kanagawa, Japan; Department of Neurosurgery, Kitasato University School of Medicine, Sagamihara, Kanagawa, Japan; Department of Neurosurgery, Kitasato University School of Medicine, Sagamihara, Kanagawa, Japan; Department of Neurosurgery, Kitasato University School of Medicine, Sagamihara, Kanagawa, Japan; Department of Neurosurgery, Kitasato University School of Medicine, Sagamihara, Kanagawa, Japan

**Keywords:** butterfly glioblastoma, corpus callosum, MGMT, surgery, survival

## Abstract

**Background:**

Glioblastoma (GB) is known for its highly invasive nature. Images of butterfly GB (bGB) often illustrate this characteristic, but the molecular background and origins of bGB remain unknown.

**Methods:**

We analyzed a cohort of 34 bGB patients from our dataset (K-cohort) and 46 bGB patients from publicly available datasets, including TCGA-GBM, CPTAC-GBM, IvyGAP, and UPENN-GBM.

**Results:**

In the K-cohort, the median age was 66 years, and molecular analyses revealed *TERT* promoter mutations in 55.9% of cases, with no cases exhibiting *H3F3A, HIST1H3B*, or *BRAF* mutations. Sequential radiological imaging from the K-cohort provided unique insights, showing one case originating in the corpus callosum (CC) and 3 cases originating in the cerebral hemisphere before developing into bGB. Multi-regional sampling supported a mutational trajectory from the hemisphere to the CC. These observations indicate the presence of 2 distinct radiological origins for bGB. Consequently, we classified cases into CC-type and Hemispheric-type based on the tumor volume ratio within the CC. This subgrouping was clinically meaningful; the CC-type is an independent poor prognostic factor for overall survival, with a hazard ratio of 1.8 (95% confidence interval 1.1–3.0, *P* = .033), and is molecularly distinct by a higher frequency of methylated *MGMT*p (*P* = .0039) compared to the Hemispheric-type.

**Conclusions:**

Our results highlight that the radiological features of bGB are not homogenous and can indicate 2 potential subtypes based on their origins. Further studies are mandatory, but CC-type and Hemispheric-type exhibit distinct clinical backgrounds, outcomes, and molecular features.

**Key Points**

- Two butterfly glioblastoma (bGB) cohorts were analyzed.
- bGB is not radiologically homogenous and can originate from either the corpus callosum (CC) or the hemisphere.
- CC-type is associated with poor outcomes and high *MGMT*p methylation.

**Importance of the Study**

The 2021 WHO classification describes bGB as “glioblastomas are usually unilateral, but they can cross the corpus callosum and be bilateral” without references or supporting evidence. This study reveals, for the first time, that bGB is not a uniform tumor entity originating solely from the hemisphere. Instead, our radiological findings suggest the possibility that bGB can also arise from the corpus callosum (CC), indicating the presence of 2 distinct subtypes based on their tumor origin: CC-type and Hemispheric-type. To validate these findings, we comprehensively analyzed 2 independent bGB cohorts, one from our institution and another from public databases. The benefit of surgical resection in bGB has been controversial in previous studies. Our analysis showed that while the CC-type is associated with a poorer prognosis, it also frequently presents *MGMT* promoter methylation, a favorable prognostic factor, which may help explain the inconsistent findings regarding the benefit of surgical resection in bGB.

Glioblastoma (GB) is a primary brain tumor with a dismal prognosis. Morphological features of GB, such as multicentric lesions,^1,2^ subventricular zone involvement,^3^ or corpus callosum (CC) involvement,^3,4^ are associated with poor survival outcomes. Butterfly glioblastoma (bGB), where GB cells invade through the CC to the contralateral hemisphere, also represents a phenotype with poor prognosis.^5–9^

Magnetic resonance (MR) images of bGB are frequently used to illustrate the symbolic features of GB, as these images well represent its highly infiltrative and malignant characteristics. Consequently, bGB appears on the cover page of the revised fourth edition of the World Health Organization (WHO) Classification of Tumors of the Central Nervous System (WHO CNS) 2016. Despite widespread knowledge of bGB imaging features, the origin and molecular characteristics remain unclear. Several studies have presented molecular data on bGB,^1,10–15^ but its molecular status is yet to be elucidated, and substantial missing data exist even in the analyzed cohort.

Early-stage GB refers to nonspecific small lesions that later progress to the classic MR imaging findings of GB, though they are clinically uncommon.^16,17^ We experienced several rare cases of early-stage GB consistent with bGB, suggesting that its origin may be in the CC or a cerebral hemisphere. Exploring the invasive trajectory of gliomas is a reasonable approach to understanding their pathophysiology, as our previous findings indicated that the parietooccipital fissure is a critical anatomical structure in glioma invasion.^18^ Given that bGB represents the most extreme phenotype of glioma invasion, we focused on its invasive trajectory. Based on observations of early-stage bGB, we hypothesized that bGB can be classified into 2 subtypes: one originating in the hemispheric and the other in the CC.

The aim of this study was to investigate the molecular features of bGB, validate the hypothesis that bGB has 2 subtypes, and elucidate clinical and molecular differences between these subtypes. To address this aim, we conducted multi-regional tumor sampling in selected cases and utilized data from public databases (TCGA-GBM,^19,20^ CPTAC-GBM,^19,21^ IvyGAP,^19,22,23^ and UPENN-GBM^24^). Understanding the tumor origin and molecular background of bGB would provide insights into its pathophysiology and guide management strategies.

## Methods

This study is a single-center retrospective study approved by the ethics committee of Kitasato University School of Medicine (IRB: G21-15). We reviewed all patients with histologically confirmed primary GB between January 2005 and April 2023. Therefore, we excluded bGB who only underwent conservative therapy without histological sampling. bGB was defined as a tumor that crosses the midline with bilateral CC involvement, as reported by Hazaymeh et al.^15^ Therefore, all bGB cases crossed at least the midline. Those with multicentric tumors with separated FLAIR high-intensity lesions were excluded. Patient demographic, pre- and postoperative Karnofsky performance status (KPS), the extent of resection (EOR), treatment, and overall survival (OS) were collected from medical records. Postoperative KPS was assessed before initiation of radiation therapy or within 1 month after surgery. The EOR was calculated using manually segmented preoperative and postoperative tumor volumes using iPlan Cranial 3.0 (BrainLab AG, Munich, Germany). The same method was used to calculate the volume of the main tumor and the tumor at the CC to obtain CC-rate {(the tumor volume at CC) × 100/the entire tumor volume)}, as reported previously.^14,15^ The tumor at the CC was anatomically delineated by the width, defined as the lateral edge of the ventricle, and the height, defined as the callosal sulcus.

### Molecular Analyses

Genomic DNA was extracted from fresh-frozen specimens using the QIAamp DNA Mini Kit (Qiagen) or from formalin-fixed paraffin-embedded (FFPE) specimens using the QIAamp DNA FFPE Tissue Kit. Hotspot mutations in *IDH1*, *BRAF*, *H3F3A*, *HIST1H3B*, and the *TERT* promoter (*TERT*p) were analyzed using Sanger sequencing. We used multiplex ligation-dependent probe amplification (MLPA) to determine copy number alterations (CNAs) using probes for *EGFR, PTEN, CDKN2A, PDGFRA, MDM2, CDK4, NFKB1A*, and *TP53* (SALSA MLPA KIT probemix P105-D2, MRC-Holland, Amsterdam, the Netherlands) as detailed in the Supplementary Materials. All the samples from multisampling cases also underwent molecular analyses. The *O*(6)-methylguanine-DNA methyltransferase promoter (*MGMT*p) methylation status was analyzed using quantitative methylation-specific PCR (qMSP) (details are in the Supplementary Materials).

### Publicly Available Dataset (Public-cohort)

For the validation cohort, we utilized publicly accessible MRI and clinical information of GB patients. Datasets were as follows: TCGA-GBM with 262 GB patients,^19,20^ CPTAC-GBM with 62 GB patients,^19,21^ IvyGAP with 41 GB patients,^19,22,23^ and UPENN-GBM with 611 GB patients,^24^ all of which included available DICOM data. Two experienced neurosurgeons (I.S. and R.S.) reviewed the DICOM data, ensuring that only cases meeting the inclusion criteria for bGB were retained. Out of the 976 cases in the validation cohort, we selected cases with sufficient data regarding age, sex, extent of resection, *IDH1* status, and *MGMT*p status. Data of TCGA-GBM and CPTAC-GBM were obtained from the cBio Cancer Genomics Portal (http://cbioportal.org)^25,26^ and GDC Data Portal (https://portal.gdc.cancer.gov/). Data of IvyGAP were obtained from the supplementary data.^23^ Data of UPENN-GBM^24^ were obtained from the publicly available repository of The Cancer Imaging Archive (TCIA)^27^ at: https://doi.org/10.7937/TCIA.709X-DN49. Some cases required to obtain idat files from GDC Data portal and assess the *MGMT*p status using DNA methylation-based classification of central nervous system tumors (https://www.molecularneuropathology.org/).^28^ Out of the total of 976 cases, 425 cases had complete data for the analysis of age, sex, surgery, *IDH1*, and *MGMT*p status, but not for KPS or exact EOR. For *MGMT*p status, cases categorized as “intermediate” were classified as “methylated.” As we calculated the CC-rate using the K-cohort, we calculated the CC-rate using DICOM data obtained from selected cases in the Public-cohort.

### Statistical Analysis

Overall survival (OS) was determined from the date of the initial surgery to the date of death or the date of the last follow-up examination. OS probability was calculated with the Kaplan–Meier method and compared with the log-rank test. Cox proportional hazards regression models with the maximum likelihood estimation were employed to identify significant independent prognostic factors for OS. Variables introduced in the multivariate analysis were chosen to ensure they are not intermediate variables^29^ between the CC-type and the outcome. The hazard ratio (HR) and a 95% confidence interval (CI) were estimated for each variable. We used JMP Pro version 16 software (SAS Institute), Prism (GraphPad Software v.8.4.3), and R 4.3.1.

## Results

### Patient Characteristics

**Figure 1.**
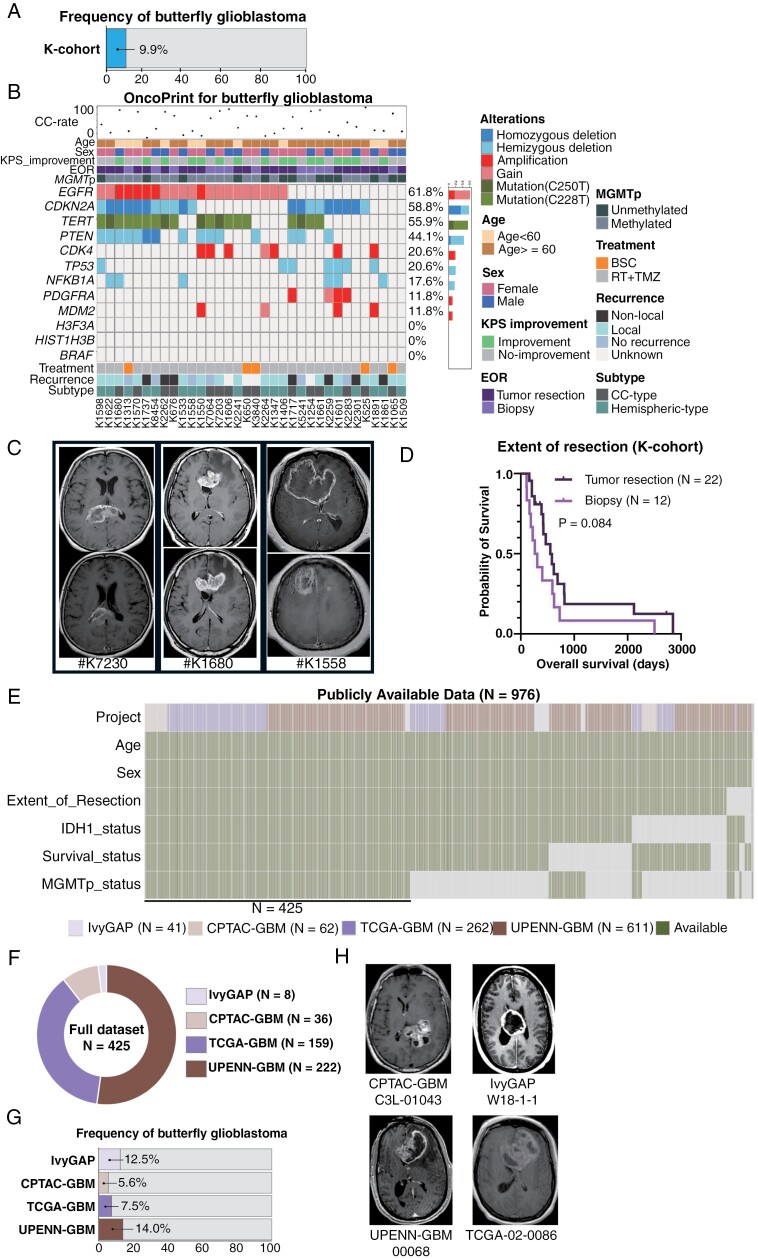
**Clinical, molecular, and radiological demographics in the Kitasato cohort (K-cohort) and publicly available data (Public-cohort).** (**A)** Frequency of butterfly glioblastoma (bGB) in the K-cohort was 9.9%. (**B)** Clinical and molecular data of all bGB cases in the K-cohort visualized by OncoPrint. The dots at the top row indicated the CC-ratio, indicating the tumor volume of the CC among the total tumor volume. The number on the right side of each figure indicates the percentage of mutated/altered cases, and the number at the bottom indicates the sample number. **(C) #K7230**: A 61-year-old female patient with bGB, presenting a deep-seated tumor. **#K1680**: A 51-year-old male patient with bGB, presenting a deep-seated tumor. **#K1558**: A 53-year-old female patient with bGB, presenting tumor portions at both the CC and hemisphere. (**D)** Kaplan–Meier curve of the overall survival (OS) based on tumor resection (*N* = 22) and biopsy (*N* = 12) in the K-cohort (*P* = .084). (**E)** 976 cases from IvyGAP, CPTAC-GBM, TCGA-GBM, and UPENN-GBM had DICOM data. Among those, age, sex, extent of resection, *IDH1* status, *MGMT*p status, and survival data were obtained from 425 cases (named as “Full dataset”). **(F and G)** Details of full dataset, and frequency of bGB among datasets. **(H)** Representative MRI from each dataset. #CPTAC-GBM: C3L-01043 and #IvyGAP: W18-1-1 presented deep-seated tumors. #UPENN-GBM: 00068 and #TCGA-02-0086 presented tumor portions at both the CC and hemisphere.

During the study period, we experienced 344 histologically confirmed GB cases, and 39 cases radiologically presented butterfly shapes. Five patients harbored *IDH1* mutation; therefore, 34 cases were included in this study and defined as K-cohort (9.9%, Figure 1A). Clinical and molecular demographics of these 34 cases are shown in Table 1 and Figure 1B. The median age of the bGB patients at diagnosis was 65 years, and 20 patients were female (58.8%). The location of CC involvement was the anterior (genu of the CC) in 15 cases (44.1%), the body of the CC in 6 cases (17.6%), and the posterior (splenium of the CC) in 13 cases (38.2%). Among the bGB patients, 13 cases (38.2%) underwent EOR ≥ 98%, 9 cases (26.5%) underwent EOR < 98%, and 12 patients underwent biopsy (35.3%). While 5 patients received the best supportive care, all others underwent 60 Gy of radiation therapy and temozolomide concurrently, followed by maintenance temozolomide therapy (the Stupp regimen).

**Table 1.** Clinical and Molecular Features of Butterfly Glioblastoma in K-cohort

		All (*n* = 34)
Age at diagnosis, median (range), years		65 (31–82)
Sex, female		20 (58.8%)
Location		
Anterior		15 (44.1%)
Body		6 (17.6%)
Posterior		13 (38.2%)
Preoperative KPS ≥ 80%		5 (14.7%)
Postoperative KPS ≥ 80%		13 (38.2%)
Extent of resection		
Resection	EOR (≥ 98%)	13 (38.2%)
	EOR (< 98%)	9 (26.5%)
	Biopsy	12 (35.3%)
*MGMT*p methylation		21 (61.8%)
*H3F3A* mutation		0 (0)
*HIST1H3B* mutation		0 (0)
*BRAF* mutation		0 (0)
*TERT*p mutation		19 (55.9%)
Copy number alteration		
* EGFR* amplification/gain		21 (61.8%)
* PTEN* hemi/homozygous deletion		15 (44.1%)
* CDKN2A* hemi/homozygous deletion		20 (58.8%)
* PDGFRA* amplification/gain		4 (11.8%)
* CDK4* amplification		7 (20.6%)
* MDM2* amplification		4 (11.8%)
* NFKBIA* hemizygous deletion		6 (17.6%)
* TP53* hemizygous deletion		7 (20.6%)
Postoperative treatment		
Radiation therapy + temozolomide		29 (85.3%)
None		5 (14.7%)
Recurrence pattern	Non-local recurrence	7 (29.2%)
	Local recurrence	17 (70.8%)
	None	6
	Unknown	4

We summarized previous studies^1,10–15,30^ involving molecular analyses of bGB. As shown in Supplementary Table 1, substantial data were missing, which limited the understanding of its molecular background. In this study, we included only histologically confirmed cases of bGB and excluded cases diagnosed merely by their typical butterfly-shaped radiological image. In our K-cohort, all patients, including those undergoing biopsy, underwent molecular analysis without any missing data. Nineteen patients (55.9%) harbored *TERT*p mutation. Biopsy cases harbored 75% of *TERT*p mutation, whereas non-biopsy cases harbored 45.5% of *TERT*p mutation (*P* = .15). Twenty-one patients (61.8%) harbored *MGMT*p methylation. Copy number alterations (CNA) of *EGFR*, *PTEN*, *CDKN2A*, and *PDGFRA* were 61.8%, 44.1%, 58.8%, and 11.8%, respectively. None harbored *H3F3A*, *HIST1H3B*, or *BRAF* mutations. Representative T1-weighted gadolinium-enhanced MRI (T1Gd) of 3 bGB cases are shown in Figure 1C. Two cases (#K7230 and #K1680) were relatively deep-seated, while the other (#K1558) presented a tumor reaching the surface of the brain. Tumor resection was associated with prolonged OS compared to biopsy, though the difference did not reach statistical significance (*P* = .084, Figure 1D). We did not include 5 *IDH*-mutated cases in the main assessments; however, for reference, we provided the MRI, clinical, and molecular findings of these butterfly astrocytoma, *IDH* mutant, WHO grade 4 (Supplementary Figure 1A–E).

### Publicly Available Dataset for the Validation Cohort

For validation, we reviewed 976 cases from publicly available GB datasets (Figure 1E) and extracted 425 cases that included age, sex, EOR, *IDH1* status, *MGMT*p status, and survival data, which we defined as the “Full dataset.” The Full dataset consisted of 159 cases from TCGA-GBM, 36 cases from CPTAC-GBM, 8 cases from IvyGAP, and 222 cases from UPENN-GBM (Figure 1F). Out of 425 patients, 49 radiologically presented with a butterfly shape; however, 3 of these patients harbored an *IDH1* mutation. Therefore, 46 cases were included and defined as the Public-cohort. The frequency of bGB was 7.5% in TCGA-GBM, 5.6% in CPTAC-GBM, 12.5% in IvyGAP, and 14.0% in UPENN-GBM (Figure 3G). Representative T1Gd of 4 bGB cases from each dataset are shown in Figure 1H. Two cases were deep-seated without any hemispheric components (the 2 cases at the top), while the other 2 cases presented with large hemispheric components extending to the surface of the brain (the 2 cases at the bottom). The location of CC involvement was the anterior in 21 cases (45.7%), the body in 11 cases (23.9%), and the posterior in 14 cases (30.4%).

### Sequential Radiological Imaging Before the Diagnosis of Typical bGB (early-stage bGB) Demonstrated the Radiological Origin

**Figure 2.**
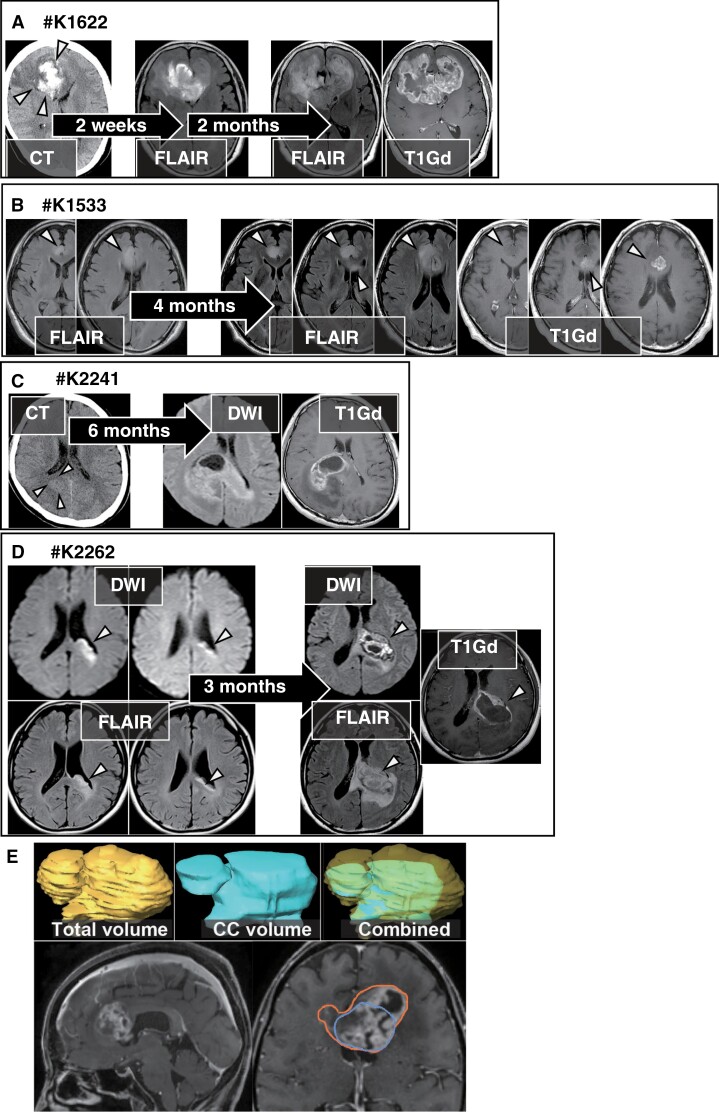
Sequential radiological imaging from early-stage GB to bGB. (**A) #K1622:** Sequential imaging of 67-year-old female patient with bGB in the Hemispheric-type. A CT imaging showed intracerebral hemorrhage at the right frontal lobe (triangles). FLAIR MRI 2 weeks later presented high intensity lesion at both the CC and contralateral frontal lobe. FLAIR and T1Gd 2 months later presented typical bGB. (**B) #K1533:** FLAIR MRI demonstrated FLAIR abnormality at the right cingulate gyrus. FLAIR and GdT1 MRI 4 months later demonstrated bGB. **(C) #K2241:** Sequential MRI of 47-year-old male patient with bGB in the Hemispheric-type. CT showed a high-density lesion in the deep white matter of the right parietal lobe (arrowheads), which 6 months later progressed to contralateral along the splenium of the CC as shown in the diffusion-weighted image (DWI) and GdT1. (**D) #K2262** Extremely rare sequential MRI, showing the CC abnormality in DWI and FLAIR. DWI, FLAIR, and GdT1 3 months later demonstrated bGB. **E:** Representative image of illustrated tumor volume and CC volume. An outer line (orange) illustrates for the total tumor and an inner (blue) line for the tumor at the CC.

Among the 34 cases in K-cohort, we experienced 4 cases of rare early-stage bGB (Figure 2A–D). A CT image of patient #K1622 revealed an intracerebral hemorrhage in the right medial frontal lobe (Figure 2A). A FLAIR MRI taken 2 weeks later showed a high-intensity lesion in the right frontal lobe and the genu of the CC, extending to the left frontal lobe. FLAIR and T1Gd MRI obtained 2 months later revealed an enhanced lesion across the bilateral frontal lobes, indicative of typical bGB (Figure 2A). FLAIR images of patient #K1533 depicted a high-intensity lesion in the right cingulate gyrus, while FLAIR and T1Gd MRI taken 4 months later showed the abnormal lesion extending to the CC and the contralateral hemisphere, representing bGB (Figure 2B). CT image of patient #K2241 depicted a slight high-density region at the deep white matter in the right parietal lobe (arrowheads), while MRI 6 months later revealed bGB (Figure 2C). Therefore, these 3 cases of early-stage bGB support the observation that hemispheric lesions can subsequently develop into bGB, as described in WHO 2021 classification of brain tumors. Conversely, diffusion-weighted images and FLAIR images of patient #K2262 showed an abnormal lesion at the CC adjacent to the subventricular zone (SVZ) without any hemispheric component. DWI, FLAIR, and T1Gd MRI 3 months later revealed the abnormal lesion extending to the splenium of the CC and toward the contralateral side, representing bGB (Figure 2D). This extremely rare case with limited hemispheric lesions demonstrates that bGB can originate directly from the CC.

### Subtyping of bGB Based on the Origin of the Tumor and Its Volume

**Figure 3.**
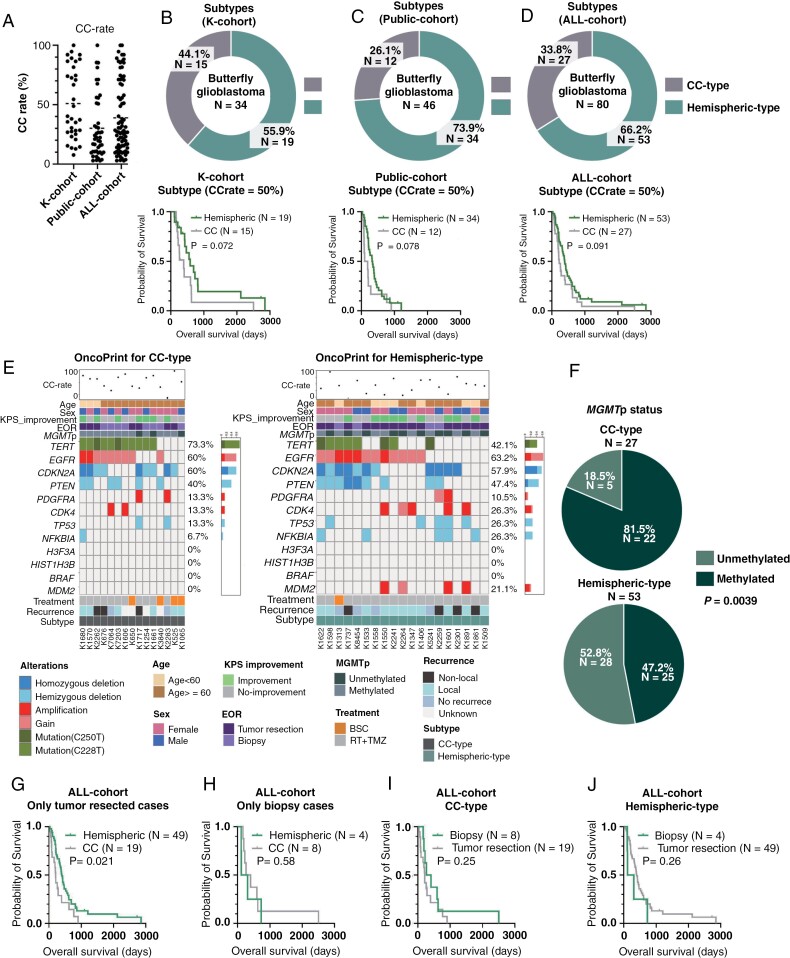
**Clinical, molecular, and survival analysis based on the CC-type and Hemispheric-type. (A)** CC-rate across the cohorts. (**B**–**D)** Pie charts illustrating the frequency of the CC-type in bGB, accompanied by Kaplan–Meier curves for OS stratified by the subtypes. (**B)** In the K-cohort, the frequency of CC-type was 44.1%, showing a trend toward poorer OS compared to the Hemispheric-type, though not statistically significant (*P* = .072). (**C)** In the Public-cohort, the frequency of CC-type was 26.1%, also showing a trend toward poorer OS compared to the Hemispheric-type (*P* = .078). **(D)** In the ALL-cohort, the frequency of CC-type was 33.8%, again showing a trend toward poorer OS compared to the Hemispheric-type (*P* = .091). (**E)** OncoPrint for the CC-type and Hemispheric-type in the K-cohort. The percentages of mutated/altered cases are indicated to the right of each figure, and the sample numbers are shown at the bottom. **(F)** The frequency of methylated and unmethylated *MGMT*p in both CC-type and Hemispheric-type in the ALL-cohort. A significantly higher prevalence of methylated *MGMT*p was observed in the CC-type (*P* = .0039). (**G**–**J)** Kaplan–Meier curve in the ALL-cohort illustrating the OS separately for tumor-resected cases (G) and biopsy cases (H) to assess the impact of subtypes, and for the CC-type (I) and Hemispheric-type (J) to assess the impact of surgery. The CC-type presented significantly poorer OS compared to the Hemispheric-type in the tumor-resected cases (*P* = .021), whereas no significant differences were observed in the others analyses.

The rare clinical experience of early-stage bGB raises the possibility that bGB can originate from the CC (Figure 2D). Indeed, radiological imaging shows variations in bGB: some cases of bGB present a small or no hemispheric component (Figure 1C: #K7230 and #K1558; Figure 1H: #C3L-01043 and #W18-101), while others present a tumor at the CC with a large hemispheric component (Figure 1C: #K1558; Figure 1H: #UPENN-GBM-00068 and #TCGA-02-0086), indicating distinct radiological patterns yet classified under the single entity of bGB. Given the extreme rarity of early-stage bGB and our experience with only one case of early-stage bGB originating from the CC, a comprehensive assessment of all bGB with an early-stage imaging was not feasible. Since the radiological features of #K2262 (Figure 2D) had limited hemispheric components, we focused on the tumor volume in the CC to estimate the possible radiological origin, CC or hemisphere. Consequently, we calculated the tumor volume of both the CC and the entire tumor^10,15^ to calculate the CC-rate, as illustrated in Figure 2E. The CC-rate was plotted in the top row of the OncoPrint (Figure 1B) and Figure 3A. The mean and median CC-rate in the K-cohort were 51.0% and 39.2%, respectively, compared to 30.1% and 21.7% in the Public-cohort. In the ALL-cohort, combining both K- and Public-cohorts, these rates were 39.1% and 28.3%, respectively. Next, we evaluated the CC rate to determine an optimal cutoff that correlates with a significant survival difference. We applied thresholds of 30%, 40%, 50%, 60%, and 70% across the K-cohort, Public-cohort, and ALL-cohorts (Supplementary Figure 2). The analyses revealed that cutoffs of 40% and 50% showed a tendency to impact survival. Also, multivariate analyses revealed that CC rates of 40% and 50% showed a significant survival impact (*P* = .025 and .011, respectively. Supplementary Table 2). Considering the need for simplicity in subgrouping, and with the 50% threshold emerging as the most suitable, we adopted a 50% cutoff. This approach allows us to differentiate bGB into 2 groups: those possibly originating from the CC (CC-type) and those possibly originating from the cerebral hemisphere (Hemispheric-type). Consequently, the K-cohort of 34 cases was subdivided into 15 cases of CC- type (44.1%, Supplementary Figure 3A-2O) and 19 cases of Hemispheric-type (55.9%, Supplementary Figure 4A-3S). Similarly, the Public-cohort included 12 cases (26.1%) and 34 cases (73.9%), and the ALL-cohort included 27 cases (33.8%) and 53 cases (66.2%) of CC- type and Hemispheric-type, respectively. All 3 cohorts similarly presented that the CC- type tended to be associated with poorer OS (Figure 3B–D, *P* = .072, .078, and .091, respectively).

### Clinical Features and Molecular Alterations in CC-type and Hemispheric-type

Clinical and molecular demographics for the CC-type and Hemispheric-type were summarized in Figure 3E, presented as an OncoPrint, and detailed in Supplementary Tables 2. In the K-cohort, when examining EOR based on subtypes, the CC-type included EOR ≥ 98% in 2 cases (13.3%), EOR < 98% in 5 cases (33.3%), and biopsy in 8 cases (53.3%), whereas the Hemispheric-type included EOR ≥ 98% in 11 cases (63.2%), EOR < 98% in 4 cases (26.7%), and biopsy in 4 cases (21.1%). These results indicate a statistically significant difference in EOR between the subtypes (*P* = .026, Supplementary Table 2). As a result, the CC-type tended to have less improvement in postoperative KPS (*P* = .079, Supplementary Table 2 and Supplementary Figure 5A). Also, the CC-type tended to have frequent *TERT*p mutations and methylated *MGMT*p compared to the Hemispheric-type, although these differences did not reach statistical significance (*P* = .079 and .092, respectively). Age, sex, and preoperative KPS showed no significant differences between the 2 subtypes (Supplementary Table 2). CNAs such as *EGFR* amplification/gain, *PTEN* hemi/homozygous deletion, and *CDKN2A* hemi/homozygous deletion also did not significantly differ between the subtypes. In the Public cohort, the CC-type presented a significantly higher frequency of *MGMT*p methylated cases compared to Hemispheric-type (*P* = .043, Supplementary Table 3). In the ALL-cohort, biopsy cases were more frequently observed in the CC-type (*P* = .017), and 81.5% of CC-type had methylated *MGMT*p, compared to 47.2% in the Hemispheric-type (*P* = .0039, Figure 3F, and Supplementary Table 3).

### Univariate and Multivariate Survival Analyses in ALL-cohort

Univariate analyses in the ALL-cohort exhibited that the median OS was 352 days (Supplementary Figure 5B). Age < 60 and methylated *MGMT*p tended to show favorable OS (*P* = .24 and .10, respectively) but not for sex (Supplementary Figure 5C–E). We conducted multivariate analyses using different CC-rates and introduced variables such as subtypes, age, sex, and *MGMT*p status, which are determined before a bGB is classified as CC-type or not, to exclude intermediate variables. Following the methodology of our univariate analyses, we employed CC-rate cutoffs of 30%, 40%, 50%, 60%, and 70%. Consistent with the findings from our univariate analysis (Figure 3B–D, and Supplementary Figure 2), CC-rates of 40% and 50% showed a significant survival impact (Supplementary Table 4). The multivariate analysis at a 50% CC-rate within the ALL-cohort revealed that the CC-type (with a HR of 1.8, 95% CI 1.1–3.0, *P* = .033) and unmethylated *MGMT*p (HR 1.8, 95% CI 1.1–3.0, *P* = .030) were independent predictors of poor OS (Table 2). For the validation, we conducted the same multivariate analyses using the K-cohort and Public-cohort, resulting in CC-type with HR 2.4 (95% CI 1.0–5.6) and HR 4.3 (95% CI 1.9–9.8), respectively (Supplementary Table 5).

**Table 2.** Multivariate Analysis of Independent Prognostic Factors Associated with Overall Survival in the ALL-cohort

Introduced variables	Hazard ratio (95%CI)	*P*-value
Subtypes		
CC-type	1.8 (1.1–3.0)	**.033**
Hemispheric-type	Ref.	
Age^*^	1.01^#^ (0.99–1.03)	.23
Sex (ref. female)	1.1 (0.69–1.8)	.69
*MGMT*p		
Unmethylation	1.8 (1.1–3.0)	**.030**
Methylation	Ref.	

^*^Continuous variables, ^#^ hazard ratio for 1 unit change, *P* values < .05 are in bold.

To exclude the crossover between subtypes and surgery, we conducted univariate analysis separately using 4 variables: biopsy, surgical resection, CC-type, and Hemispheric-type. First, we assessed the importance of the subtypes separately in surgically resected and biopsied cases. In the resected cases, the CC-type was associated with poorer OS (Figure 3G, *P* = .021), whereas, in the biopsied cases, no significant differences were observed between CC-type and Hemispheric-type (Figure 3H, *P* = .58). Next, within the CC-type, although there were no statistical differences, biopsy cases tended to show longer OS (Figure 3I). In the Hemispheric-type, while not statistically significant, tumor-resected cases tended to show longer OS (Figure 3J).

Moreover, introducing the CC-rate as a continuous variable in the tumor-resected cases revealed that the CC-rate is an independent predictor of poor OS (HR 1.01, 95% CI 1.0–1.02, *P* = .030, Supplementary Table 6). Univariate analyses for the anterior, body, and posterior involvement of the CC did not show a difference (Supplementary Figure 5F–G). Butterfly astrocytoma, *IDH* mutant, grade 4 showed significantly favorable OS compared to bGB (*P* = .0085, Supplementary Figure 1D).

### Multi-regional Sampling to Investigate the Molecular Evolutionary Trajectory of bGB

**Figure 4.**
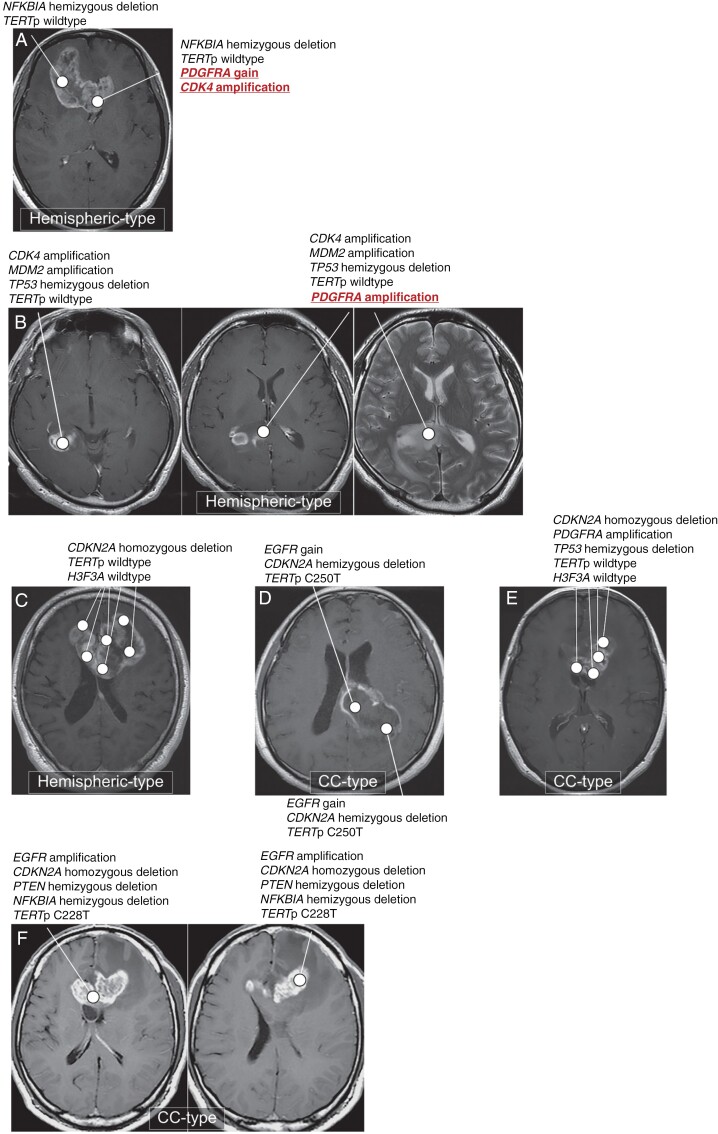
MRI and molecular data from multi-regional sampling in CC-type and Hemispheric-type. (**A)** #K1861 in the Hemispheric-type. The hemispheric tumor presented a *NFKBIA* hemizygous deletion and *TERT*p wildtype. The CC tumor shared these molecular alterations and had additional *PDGFRA* gain and *CDK4* amplification. (**B**) #K1891 in the Hemispheric-type. The hemispheric tumor presented *CDK4* amplification*, MDM2* amplification, *TP53* hemizygous deletion, and *TERT*p wildtype. The CC tumor shared these molecular alterations and had additional *PDGFRA* amplification. (**C**–**F**) Multi-regional sampling cases in the Hemispheric-type (**C:** #K2301) and the CC-type (**D:** #K2262, **E:** #K2283, and **F:** #K1680), sharing the same alterations across various tumor locations. D: #K2262 represents an early-stage bGB of the CC-type, as also referenced in Figure 2D.

In the K-cohort, 6 patients (Figure 4A–F), including 3 Hemispheric-types (4A-C) and 3 CC-types (Figure 4D–F), underwent multi-regional sampling. Two cases presented additional alterations in the CC region compared to the hemispheric region. Specifically, the case shown in Figure 4A had a *NFKBIA* hemizygous deletion and *TERT*p wildtype in the hemispheric region, whereas the CC region had additional *PDGFRA* gain and *CDK4* amplification. Similarly, the case in Figure 4B indicated that the CC region had additional *PDGFRA* amplification compared to the hemispheric region. These 2 cases (Figure 4A and 4B), categorized as Hemispheric-type based on the CC-rate, likely originated from the cerebral hemisphere, as supported by the molecular evolutionary trajectories obtained from multi-regional sampling. In contrast, another case of Hemispheric-type (Figure 4C) and 3 cases of CC-type (Figure 4D–F) exhibited no differences in major molecular alterations among the specimens analyzed through multi-regional sampling. Figure 4D corresponds to the case with an early-stage bGB indicative of CC-origin, as shown in Figure 2D. A case of butterfly astrocytoma, *IDH* mutant, grade 4 also underwent multi-regional sampling (Supplementary Figure 1B).

## Discussion

This study investigated both the radiological and molecular characteristics of bGB. While many previous studies lacked comprehensive molecular analyses of bGB, we assessed major and frequent molecular alterations to better understand the molecular background of bGB. The WHO 2021 classification generally describes bGB as originating from the unilateral hemisphere and subsequently invading the CC. However, our observation of an early-stage bGB case located in the CC raises the possibility that bGB might, in some cases, originate within the CC. The imaging features of bGB are diverse; some cases have large hemispheric components, while others have minimal or no hemispheric components. Therefore, assuming that all bGB cases arise exclusively from the cerebral hemisphere may not fully capture the complexity of this tumor type. This study offers additional insights into the diverse radiological and pathophysiological characteristics of bGB.

### Subdividing bGB into CC-type and Hemispheric-type Enables the Identification of Distinct Prognostic Variations

Although bGB is generally seen as having a poor prognostic, our findings reveal that the CC-type has an even worse prognostic. While univariate analyses suggested a trend toward poorer prognosis for the CC-type compared to the Hemispheric-type, this observation was conclusively confirmed through multivariate analyses.

One primary factor contributing to the poor prognosis in the CC-type may be related to clinical approaches, particularly the EOR and postoperative treatments. Our K-cohort exhibited a tendency toward less aggressive resection and postoperative treatments in the CC-type. Similarly, a study by Bjorland et al., which investigated deep-seated bGB, revealed that this subtype was associated with poorer OS in both univariate and multivariate analyses.^14^ They defined deep-seated locations as tumors mainly located in the thalamus, basal ganglia, capsula interna, splenium of the corpus callosum, or mesencephalon. Despite differences in classification methods, both their deep-seated bGB and our CC-type bGB highlight similar challenges in treatment decisions. Hemispheric-type, or non-deep-seated tumors, typically have a superficial component allowing for easier access, enabling neurosurgeons to perform resections without hesitation. In contrast, CC-type tumors are covered by intact brain tissue, making them less accessible for surgical resection without risking damage to healthy brain areas. Consequently, surgical resections for CC-type are not as technically simple as those for cerebral GB or Hemispheric-type. Also, our data suggested that postoperative KPS in the CC-type tended to be lower than that in the Hemispheric-type. This finding aligns with a study by Dayani et al., indicating that postoperative KPS in bGB generally does not improve, not necessarily due to surgical deficits.^10^ Generally, postoperative KPS in GB cases improves in approximately 57% of cases, remains stable in about 25%, and declines in only 18% of cases.^31^ Therefore, the postoperative course of CC-type is not as straightforward as what is usually observed in cerebral GB.

Second, the pathophysiology of the CC-type may contribute to its poor prognosis. To exclude the effect of EOR, we assessed survival differences between the subtypes separately for those who underwent tumor resection and those who had a biopsy. Univariate analysis showed that the CC-type was associated with significantly poorer OS compared to the Hemispheric-type in tumor-resected cases, but not in biopsy cases. In addition, to determine whether a higher CC-rate affects OS among tumor-resected cases, we conducted a multivariate analysis and found that a higher CC-rate was indeed associated with poorer OS. Thus, less aggressive EOR alone does not fully explain the poor OS observed in the CC-type. Although several cases were lost to follow-up, leading to unknown recurrence patterns, certain CC-type cases exhibited non-local recurrence. A distinctive feature of the CC-type is the alignment of the entire tumor along the SVZ, with SVZ involvement being a known factor contributing to non-local recurrence.^32^ Therefore, it is plausible that the CC-type behaves physiologically differently from the Hemispheric-type, potentially leading to a higher risk of non-local recurrence and poorer prognosis.

### Impact of Surgical Resection on Survival

Despite extensive discussion in the literature,^1,6,7,9–11,14,33–36^ no consensus has been reached on the benefit of surgical resection for bGB. Previous studies on EOR in bGB, such as those by Chaichana et al.^6^ and Dayani et al.,^10^ reported survival benefits at EOR thresholds of 65% and 86%, respectively. In contrast, Bjorland et al. found no significant survival difference between bGB cases that underwent surgical resection and those that did not.^14^ Our study raises the possibility that the subtypes may influence surgical outcomes, potentially explaining the inconsistencies and lack of consensus in the literature. Notably, the distribution of subtypes varies across cohorts: the K-cohort included 44.1% of CC-type, while the Public-cohort included 26.1%. In the public database, the frequency of CC-type was 8.3% in TCGA-GBM (one out of 12 cases), 0% in CPTAC-GBM (0 out of 2 cases), 100% in IvyGAP (one out of one case), and 32.2% in UPENN-GBM (10 out of 31 cases). To address these discrepancies, we separately assessed the prognostic impact of bGB using 4 variables: biopsy, surgical resection, CC-type, and Hemispheric-type. Although the analyses were limited by sample size, we found that the CC-type was associated with poorer OS, particularly in cases undergoing tumor resection. These findings suggest that the varied frequency of CC-type across studies may obscure the actual prognostic impact of EOR in bGB and these factors need to be carefully considered in future studies.

Existing evidence suggests that 50% of patients with high-grade glioma who only receive a biopsy fail to complete radiation therapy, primarily due to deteriorating KPS.^5^ Moreover, the National Cancer Database, a nationwide database curated by the American College of Surgeons’ Commission on Cancer, reveals that 59.1% of bGB patients undergo a biopsy, compared to 28.6% of non-bGB patients, and are less likely to receive subsequent chemotherapy and radiotherapy.^1^ This suggests that the survival outcomes in bGB patients are strongly affected by biopsy cases, which often leads to less frequent chemo/radiotherapy. Recently, Dadario et al. presented the endoscope-assisted removal for bGB^30^ and Daggubati et al. presented bilateral laser interstitial thermal therapy for bGB.^37^ We presented bilateral trans-sulcus and interhemispheric approaches for anterior bGB, especially for CC-type.^38^ All 3 studies have tried to surgically treat bGB while minimizing the damage to the unaffected brain. Therefore, tailoring the surgical approach to enable the completion of subsequent chemo/radiotherapy may be sufficient to improve the prognosis for CC-type. As Soliman et al. described in their meta-analysis, there is a trend toward resection for bGB but the high rate of postoperative deficits remains a significant concern.^36^ Future studies should assess the feasibility of aggressive resection for bGB through a precise neuropsychological examination conducted both preoperatively and postoperatively.

### Molecular Alterations of bGB, Subgrouping, and Their Impact on Survival

We hypothesized that bGB is a distinct subtype of GB, characterized by an aggressive phenotype due to accumulated molecular alterations. This hypothesis has not been adequately addressed in the current literature, as substantial molecular data were missing in previous reports (Supplementary Table 1).^1,10–15,30^ Our K-cohort, which had no missing data, provided a comprehensive overview of the genetic landscape of bGB. The *TERT*p mutation was detected in 55.9% of bGB cases. While the range of *TERT*p mutation in general GB ranges from 55% to 80.3%,^39–42^ the rate in bGB was not notably high. Similarly, CNA of *EGFR*, *PTEN*, and *CDKN2A* in bGB were 61.8%, 44.1%, and 58.8%, respectively. Although these alterations can exhibit regional differences,^42^ the rates in bGB were not notably higher than those observed in general GB.^42^ Therefore, bGB does not exhibit a substantial accumulation of major molecular alterations compared to general GB. A previous report suggested that *IDH-*wildtype *TERT*p-mutated glioblastoma can exhibit aggressive clinical and radiological phenotypes.^39^ In this study, radiologically diagnosed bGB, which may harbor *TERT*p mutations, was excluded, leading to uncertainty about the actual frequency of *TERT*p mutations in bGB. This exclusion may explain the relatively lower rate of *TERT*p mutations observed in bGB. Furthermore, the higher frequency of *TERT*p mutations in CC-type and biopsy cases in our K-cohort can also be attributed to these aggressive phenotypes.

Upon subgrouping bGB, we radiologically divided bGB into 2 subtypes: CC-type and Hemispheric-type, which showed meaningful prognostic differences. Methylated *MGMT*p was significantly more frequent in CC-type than in Hemispheric-type, supporting this subgrouping from a molecular perspective. Chojak et al.,^34^ Boaro et al.,^1^ and Franco et al.^11^ reported that 60%, 57.1%, and 64.7% of bGB cases undergoing biopsy, respectively, had methylated *MGMT*p. In our K-cohort, the CC-type, characterized by its deep-seated location, tended to undergo biopsy and showed a higher prevalence of methylated *MGMT*p. Therefore, their findings on biopsy cases support our findings in the CC-type, suggesting similar clinical and molecular features. We experienced a case of CC-type that underwent biopsy and was treated with the Stupp regimen, surviving for 6 years. This case (#K1606, as shown in Supplementary Figure 3B) had methylated *MGMT*p and responded significantly to TMZ. Our observations align with Bjorland’s study,^14^ which also included biopsy cases achieving an OS of over 2 years. The high prevalence of methylated *MGMT*p in CC-type suggests that completing the Stupp regimen may improve survival outcomes for patients with this subtype.

Another significant aspect of our study was the results from multi-regional sampling of bGB. This approach allowed us to validate that the mutational trajectory progresses from the hemisphere to CC in the Hemispheric-type. However, we did not observe a trajectory from the CC to the hemisphere in the CC-type, which may be due to our molecular analysis being limited to only major molecular alterations. Therefore, a more comprehensive molecular analysis, including next-generation sequencing with variant allele frequency, could provide insights into specific molecular alterations that differentiate CC-type from Hemispheric-type, or uncover a mutational trajectory from the CC to the hemisphere.

### What Is the Origin of bGB?

It has been traditionally believed that bGB originates in the cerebral hemisphere and then infiltrates the contralateral hemisphere via the CC. In the case shown in Figure 4A, it is not apparent whether the tumor in the CC invaded toward the cerebral hemisphere or if the tumor in the cerebral hemisphere invaded toward the CC. In this specific case, our multisampling approach provided evidence supporting the idea that the trajectory is from the hemisphere to the CC. Also, our examination of early-stage bGB cases and multi-regional sampling cases supported the idea that the predominant invasive trajectory is from the hemisphere to the CC. Galldiks et al. reported a posterior bGB case using methionine (MET) positron emission tomography, revealing an uneven distribution of MET uptake.^43^ While a detailed radiological assessment was not feasible from the published image, the distribution of the tumor was characterized by a limited cerebral hemispheric lesion resembling CC-type. The MET uptake was higher in the left side of the splenium of the CC and gradually diminishing in the surrounding structures. Given the correlation between higher tumor cell density and elevated MET uptake,^44^ the Galldiks case also supports that the CC as a potential origin of bGB.

bGB occasionally presents completely symmetrical bilateral extension or a primary tumor location within the CC, which cannot be solely explained by the hemisphere-to-CC invasive trajectory. Fortunately, we have obtained evidence from an early-stage bGB case that confirmed its origin at the CC. Anatomically, the CC consists of a bundle of fiber tracts, including approximately 200 million axons,^45^ crossing from both hemispheres. Previous evidence has suggested the existence of 4 midline glial populations (glial wedge, indusium griseum, midline zipper, and the glial sling) within the CC.^45,46^ In addition, GFAP-positive astrocytes have been identified in the CC.^47^ Therefore, these observations suggest that the CC could serve as an origin for gliomas.

The midline location of the CC and the typically poor prognosis associated with bGB have raised questions about whether bGB is an H3 K27-altered diffuse midline glioma. Two previous studies have addressed this question.^1,13^ Boaro et al. immunohistochemically analyzed 5 out of 62 bGB patients and all were found to be wildtype.^1^ Paoli et al. investigated 49 cases of bGB and found no evidence of *H3F3A* and *HIST1H3B* mutations through immunohistochemistry and pyrosequencing.^13^ Our own data align with these findings, as the bGB patients in our cohort also did not harbor mutations indicative of diffuse H3 K27-altered midline gliomas. Therefore, bGB is not a diffuse midline glioma, and the poor prognosis of bGB patients is not attributed to diffuse midline gliomas. However, it is important to note that we cannot exclude the possibility of midline gliomas originating from the CC. In addition, there have been reports of several midline gliomas with *H3K27M* mutations originating from the CC.^48–50^ Therefore, bGB should not be classified as a midline glioma, but it is important to acknowledge that a small number of midline gliomas can indeed develop within the CC and exhibit a butterfly shape.

### Limitations of this Study

This study has several limitations. First, the rarity of bGB and the retrospective single institutional nature of the study limited the number of patients included. To exclude these limitations, we obtained a fully molecularly analyzed dataset and established a validation cohort using publicly available data. Both cohorts presented similar results, enhancing the robustness of our findings. However, the sample size was still not sufficient to assess the impact of surgical resection and subtypes separately, so further studies with larger cohorts are necessary. Second, we had only one case of early-stage bGB of the CC-type. To address this rarity, we categorized bGB into CC-type and Hemispheric-type based on the tumor volume at the CC. While this grouping may not perfectly capture the true origin of bGB, it allowed us to explore the possibility that bGB can be subdivided into different types, which may have implications for survival differences. Third, we only conducted direct sequencing to assess bGB. Further molecular analyses, such as single-cell or next-generation sequencing, could provide validation and deeper insights into our findings.

## Conclusions

The classic understanding of GB progression suggests that when a tumor is present in both the cerebral hemisphere and CC, the CC portion likely originated from the hemispheric portion. However, some bGB cases exhibit minimal or no hemispheric component, suggesting classic understanding may not fully explain the radiological heterogeneity of bGB. We examined images of early-stage bGB, major molecular alterations in bGB, and findings from multiregional samplings, suggesting that bGB might originate from either the CC or the hemisphere. While the CC-type appears to have poorer clinical outcomes among bGB cases, the high prevalence of methylated *MGMT*p in this subtype suggests there may be potential for improved survival through optimized surgery and postoperative treatments.

## Supplementary Material

vdae180_suppl_Supplementary_Figure_S1

vdae180_suppl_Supplementary_Figure_S2

vdae180_suppl_Supplementary_Figure_S3

vdae180_suppl_Supplementary_Figure_S4

vdae180_suppl_Supplementary_Figure_S5

vdae180_suppl_Supplementary_Materials

vdae180_suppl_Supplementary_Tables_S1-S6

## Data Availability

Dataset may be available upon request.

## AuthorshipStatement

Conception and design: I.S.; Acquisition of data: I.S., R.S., Y.T., K.F., H.H., Y.H., M.T., M.I., K.U., M.S., H.K., S.S., T.H.; Analysis and interpretation of data: I.S., R.S., T.W., Y.O., Drafting the article: I.S., Y.O., K.F., T.K., Manuscript review: all authors.
