# Supplementary figures and images for: Radiological, clinical, and molecular analyses reveal distinct subtypes of butterfly glioblastomas affecting the prognosis
Source: Neurooncol Adv. 2024 Oct 23;6(1):vdae180. doi: 10.1093/noajnl/vdae180 (PMC11647517; doi:10.1093/noajnl/vdae180)

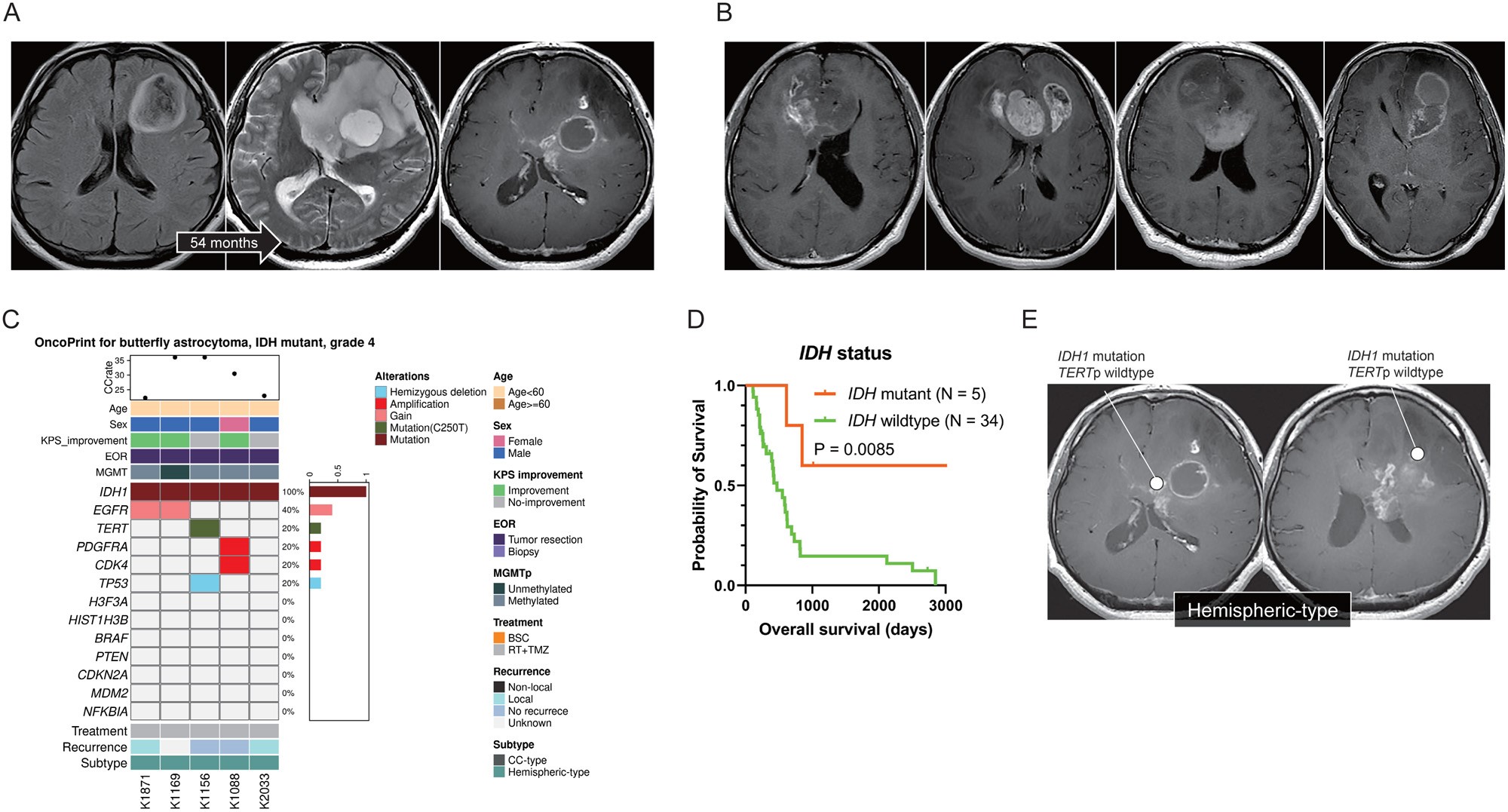

Supplement: vdae180_suppl_Supplementary_Figure_S1 [file vdae180_suppl_supplementary_figure_s1.jpeg]

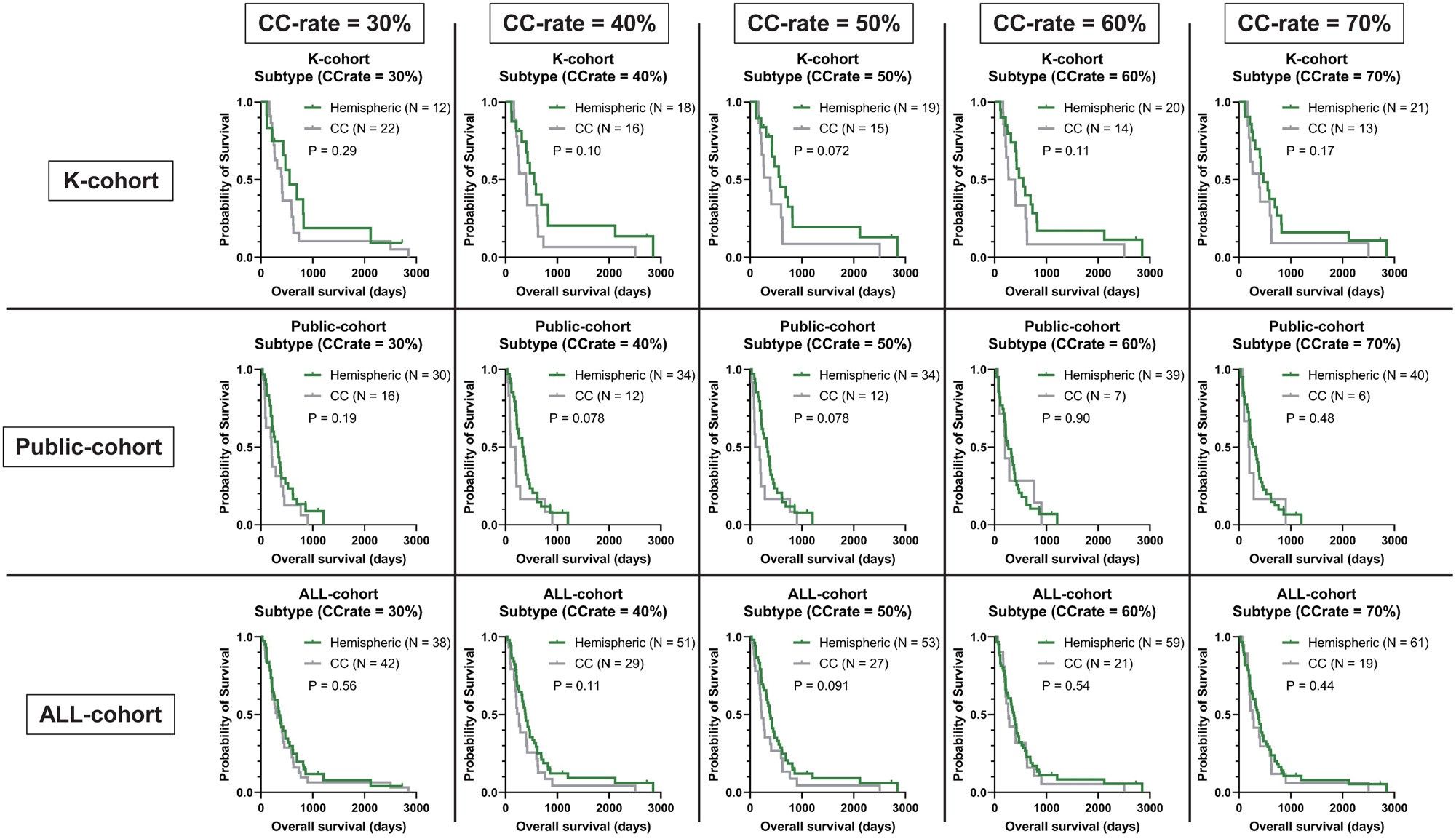

Supplement: vdae180_suppl_Supplementary_Figure_S2 [file vdae180_suppl_supplementary_figure_s2.jpeg]

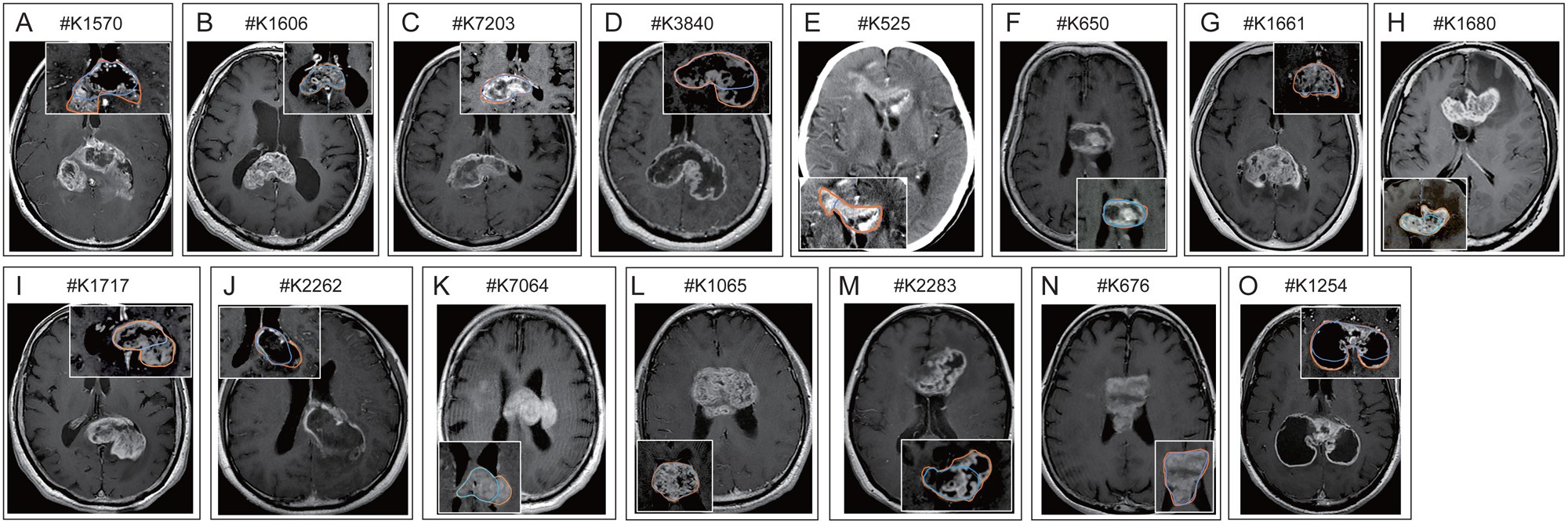

Supplement: vdae180_suppl_Supplementary_Figure_S3 [file vdae180_suppl_supplementary_figure_s3.jpeg]

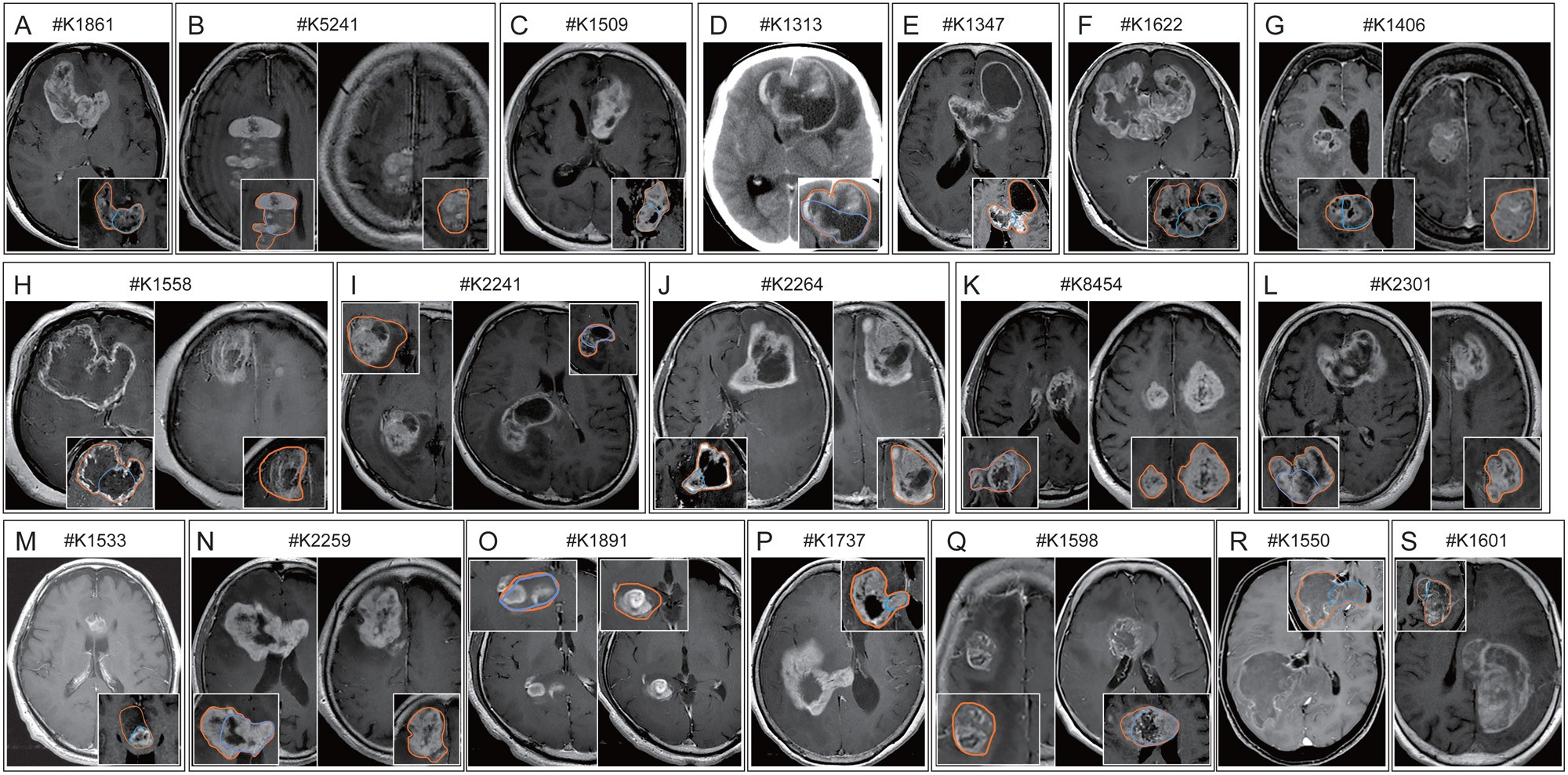

Supplement: vdae180_suppl_Supplementary_Figure_S4 [file vdae180_suppl_supplementary_figure_s4.jpeg]

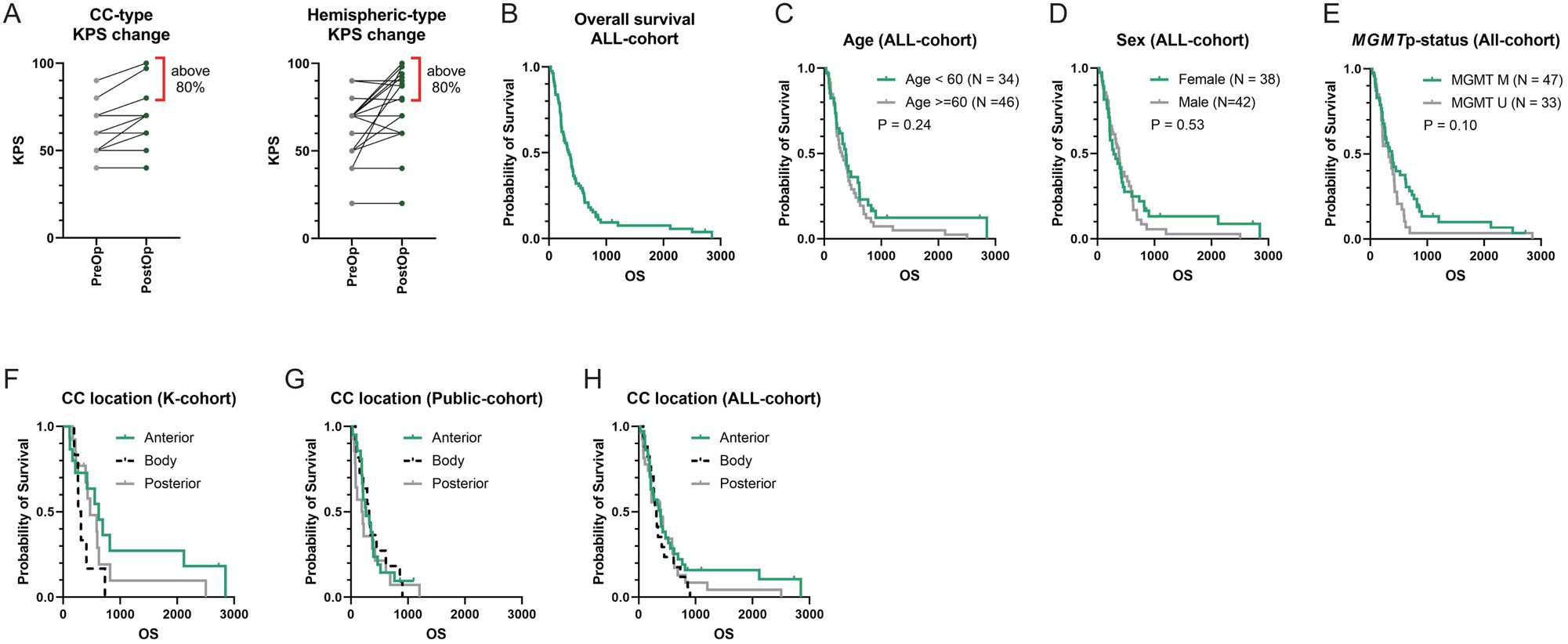

Supplement: vdae180_suppl_Supplementary_Figure_S5 [file vdae180_suppl_supplementary_figure_s5.jpeg]
