# Supplementary material for: Radiological, clinical, and molecular analyses reveal distinct subtypes of butterfly glioblastomas affecting the prognosis
Source: Neurooncol Adv. 2024 Oct 23;6(1):vdae180. doi: 10.1093/noajnl/vdae180 (PMC11647517; doi:10.1093/noajnl/vdae180)
Supplement: vdae180_suppl_Supplementary_Materials [file vdae180_suppl_supplementary_materials.docx]

**Molecular analyses**

Genomic DNA was extracted from fresh-frozen specimens using the QIAamp DNA Mini Kit (Qiagen) or from formalin-fixed paraffin-embedded (FFPE) specimens using the QIAamp DNA FFPE Tissue Kit. Hotspot mutations in *IDH1*, *BRAF*, *H3F3A*, *HIST1H3B*, and the *TERT* promoter (*TERT*p) were analyzed using Sanger sequencing. We used multiplex ligation-dependent probe amplification (MLPA) to determine copy number alterations (CNAs) using probes for *EGFR, PTEN, CDKN2A, PDGFRA, MDM2, CDK4, NFKB1A*, and *TP53* (SALSA MLPA KIT probemix P105-D2, MRC-Holland, Amsterdam, the Netherlands). The thresholds of copy number detection were above 1.2 for gain, below 0.8 for loss, below 0.4 for homozygous deletion, and above 2.0 for amplification.^1,2^ Noncanonical CNAs, such as amp/gain in *CDKN2A*, *PTEN*, *TP53*, or *NFKBIA* and deletion in *PDGFRA*, *EGFR*, *CDK4*, or *MDM2,* were not considered in the data analysis, as previously described.^3^ All the samples from multisampling cases also underwent molecular analyses.

**Quantitative methylation-specific PCR (qMSP) for *MGMT*p methylation**

The *O*(6)-methylguanine-DNA methyltransferase promoter (*MGMT*p) methylation status was analyzed using quantitative methylation-specific PCR (qMSP).^4^

Bisulfite conversion of 400 ng of genomic DNA per sample was conducted using the EZ DNA Methylation-Gold Kit (Zymo Research Corporation). qPCR was conducted on 40 ng of bisulfite-converted genomic DNA using 0.4 μM each of the forward and reverse primers, 0.2 μM of the probe and 1x Luna Universal Probe qPCR Master Mix (NEB) in a final volume of 10 μl. Two different probes were designed specifically for methylated or unmethylated DNA molecules. The 5’ ends of methylated and unmethylated *MGMT* probes were labeled with a FAM or HEX fluorochrome, respectively. Both probes have a quencher with a minor groove binder (MGB) at the 3’ end. The thermal cycling included an initial step at 95℃ for 1 minute followed by 42 cycles of 15 seconds at 95℃, 20 seconds at 55℃, and 20 seconds at 65℃. The percentage of methylated *MGMT* DNA was calculated for each sample from doublets by dividing the methylated *MGMT* copy numbers in the target by the total of methylated plus unmethylated *MGMT* copy numbers and multiplying by 100 [Met/(Met+UnMet) *100]. Reactions were conducted three times, and the percentage averages were calculated.

We used the cutoff value of 1.0% to determine the methylation status (above 1.0%, methylated; below 1.0%, unmethylated) as previously described.^3^

The *MGMT* qMSP primers and probe for detecting methylated sequences were as follows:

Forward primer: 5’- TTTCGACGTTCTAGGTTTTCGC -3’

Reverse primer 5’- GCACTCTTCCGAAAACGAAACG -3’

Probe 5’- [FAM] CAAATCGCAAACGATA [MGBEQ] -3’

The *MGMT* qMSP primers and probe for detecting unmethylated sequences were as follows:

Forward primer 5’- TTTGTGTTTTGATGTTTGTAGGTTTTTGT -3’

Reverse primer 5’- AACTCCACACTCTTCCAAAAACAAAACA -3’

Probe 5’-[HEX] CAAATCACAAACAATA [MGBEQ] -3’

Complex heatmaps were used to visualize genomic alterations as OncoPrint.^5^

**1.** Makino Y, Arakawa Y, Yoshioka E, et al. Prognostic stratification for IDH-wild-type lower-grade astrocytoma by Sanger sequencing and copy-number alteration analysis with MLPA. *Sci Rep.* 2021; 11(1):14408.

**2.** Shibahara I, Sonoda Y, Saito R, et al. The expression status of CD133 is associated with the pattern and timing of primary glioblastoma recurrence. *Neuro Oncol.* 2013; 15(9):1151-1159.

**3.** Umehara T, Arita H, Yoshioka E, et al. Distribution differences in prognostic copy number alteration profiles in IDH-wild-type glioblastoma cause survival discrepancies across cohorts. *Acta Neuropathol Commun.* 2019; 7(1):99.

**4.** Rosas-Alonso R, Colmenarejo-Fernandez J, Pernia O, et al. Clinical validation of a novel quantitative assay for the detection of MGMT methylation in glioblastoma patients. *Clin Epigenetics.* 2021; 13(1):52.

**5.** Gu Z, Eils R, Schlesner M. Complex heatmaps reveal patterns and correlations in multidimensional genomic data. *Bioinformatics.* 2016; 32(18):2847-2849.
