# Supplementary material for: Radiological, clinical, and molecular analyses reveal distinct subtypes of butterfly glioblastomas affecting the prognosis
Source: Neurooncol Adv. 2024 Oct 23;6(1):vdae180. doi: 10.1093/noajnl/vdae180 (PMC11647517; doi:10.1093/noajnl/vdae180)
Supplement: vdae180_suppl_Supplementary_Tables_S1-S6 [file vdae180_suppl_supplementary_tables_s1-s6.docx]

**Supplementary Figure 1** Radiological, clinical, and molecular features of five cases with butterfly astrocytoma, *IDH* mutant, grade 4. **A:** Sequential imaging of a 49-year-old male patient with bGB in the Hemispheric-type. A FLAIR imaging showed a high-intensity region at the left frontal lobe. T2 and GdT1 MRI 54 months later presented typical butterfly astrocytoma. **B:** Four other GdT1 images of butterfly astrocytomas. **C:** OncoPrint for the five butterfly astrocytomas showing clinical and molecular data. The percentages of mutated/altered cases are indicated to the right of each figure. **D:** A Kaplan‒Meier curve comparing the OS of five cases of butterfly astrocytoma, *IDH* mutant, grade 4 versus bGB in the K-cohort, suggesting a significant favorable prognosis in the former (P = 0.0085). **E:** Multi-regional sampling of the case in Supplementary Figure 1A. The hemispheric and CC tumors shared molecular alterations of *IDH1* mutation and *TERT*p wildtype.

**Supplementary Figure 2** Kaplan‒Meier curves comparing the OS of CC-type versus Hemispheric-type, analyzed according to the CC-rate within the K-cohort, Public-cohort, and ALL-cohort. CC-rates of 30%, 40%, 50%, 60%, and 70% were employed to examine their influence on OS across the three cohorts.

**Supplementary Figure 3 A-O:** Radiological imaging of the CC-type in the K-cohort, with manual segmentation highlighted by an orange line for the total tumor and a blue line for the tumor at the CC.

**Supplementary Figure 4 A-S:** Radiological imaging of the Hemispheric-type in the K-cohort, with manual segmentation highlighted by an orange line for the total tumor and a blue line for the tumor at the CC. In selected cases, two images are shown to demonstrate the presence of a large hemispheric tumor component.

**Supplementary Figure 5** **A:** Changes between pre- and postoperative KPS assessed separately in the CC-type and Hemispheric-type. **B-E:** Univariate analyses in the ALL-cohort. OS of the entire cohort (**B**), age (**C**), sex (**D**), and *MGMT*p status (**E**). **F-H:** Univariate analyses among tumor involvement of the CC location in the K- (**F**), Public- (**G**), and ALL-cohorts (**H**). Comparison among the anterior (the genu of the CC), body, and posterior portion (the splenium of the CC).

**Supplementary Table 1** Summary of previous studies reporting molecular alterations of bGB

| Author & Year | No. of patients | *TERT*p | *H3F3A* | *HIST1H3B* | *BRAF* | *IDH1* | *EGFR* | *PTEN* | *CDKN2A* | *PDGFRA* | *TP53* | *MGMT*p |
| --- | --- | --- | --- | --- | --- | --- | --- | --- | --- | --- | --- | --- |
| Hazaymeh  2022 | bGB N=27 | NA | NA | NA | NA | Unknown  mut: 31%  mis.: 11 | NA | NA | NA | NA | IHC  pos.: 69%  mis.: 11 | Unknown  met: 38%  mis.: 11 |
| Dayani 2018 | bGB N=39 | NA | NA | NA | NA | Unknown  mut: 7.7%  mis.: 26 | Unknown  amp: 47%  mis.: 22 | Unknown  del: 36.4%  mis.: 28 | NA | NA | Unknown  mut: 50%  mis.: 31 | Unknown  met: 20%  mis.: 34 |
| Opoku-Darko  2018 | bGB N=29 | NA | NA | NA | NA | Unknown  mut: 0%  mis.: 20 | NA | NA | NA | NA | NA | Unknown met: 50%  mis.: 21 |
| Paoli  2021 | bGB N=49 | NA | Pyro&IHC  mut: 0% | Pyro&IHC  mut: 0% | NA | NGS  mut: 0% | NA | NA | NA | NA | NA | Pyro  met: 39.5% |
| Boaro  2021 | bGB N=62 | NA | IHC  mut: 0%  mis.: 57 | NA | NA | IHC  mut: 3.8%  mis.: 10 | FISH  amp: 29.4% mis.: 11 | NA | NA | NA | NA | MSP met:55.6%  mis.: 17 |
| Bjorland  2022 | bGB N=33 | NA | NA | NA | Seq  mut: 0%  mis.: 22 | IHC:  mut: 0% | NA | NA | NA | NA | NA | qMSP  met: 18%  mis.: 22 |
| Franco  2021 | bGB N=55 | NA | NA | NA | NA | IHC+NGS  0% | NA | NA | NA | NA | NA | Unknown  met: 50%  mis.: 25 |
| Dadario  2022 | bGB N=70 | NA | NA | NA | NA | IHC  mut: 25%  mis.: 26 | IHC  pos.: 48%  mis.: 44 | NA | NA | NA | IHC  pos.: 61%  mis.: 28 | NA |
| Present series | bGB N=34 | Seq  mut: 55.9% | Seq  mut: 0% | Seq  mut: 0% | Seq  mut: 0% | Seq  mut: 0% | MLPA  amp: 61.8% | MLPA  del: 44.1% | MLPA  del: 58.8% | MLPA  amp: 11.8% | MLPA  del: 20.6% | qMSP  met: 61.8% |

Underline indicates the method; NA, not applicable; IHC, immunohistochemistry; mut, mutation; mis, missing; pos, positive; FISH, fluorescence in situ hybridization; MSP, methylation-specific polymerase chain reaction; Pyro, pyrosequencing; NGS, next-generation sequence; met, methylated; amp, amplification; del, deletion; Seq, sequencing; MLPA, multiplex ligation-dependent probe amplification; qMSP, quantitative MSP

**Supplementary Table 2 Clinical and molecular features of butterfly glioblastoma in K-cohort based on the subtypes**

|  | | K-cohort | | |
| --- | --- | --- | --- | --- |
|  | | CC-type (n = 15) | Hemispheric-type (n = 19) | P-value^a^ |
| Age at diagnosis, median (range), years | | 69 (48-76) | 54 (31-82) | 0.57 |
| Sex, female | | 9 (60) | 11 (57.9) | 1.0 |
| Location | |  |  |  |
| Anterior | | 3 (20) | 12 (63.2) | 0.041 |
| Body | | 4 (26.7) | 2 (10.5) |  |
| Posterior | | 8 (53.3) | 5 (26.3) |  |
| Preoperative KPS ≥ 80% | | 2 (13.3) | 3 (15.8) | 1.0 |
| Postoperative KPS ≥ 80% | | 3 (20) | 10 (52.6) | 0.079 |
| Extent of resection | |  |  |  |
| Resection | EOR (≥ 98%) | 2 (13.3) | 11 (63.2) | **0.026** |
|  | EOR (< 98%) | 5 (33.3) | 4 (26.7) |  |
|  | Biopsy | 8 (53.3) | 4 (21.1) |  |
| *MGMT*p methylation | | 12 (80) | 9 (47.3) | 0.079 |
| *H3F3A* mutation | | 0 (0) | 0 (0) | 1.0 |
| *HIST1H3B* mutation | | 0 (0) | 0 (0) | 1.0 |
| *BRAF* mutation | | 0 (0) | 0 (0) | 1.0 |
| *TERT*p mutation | | 11 (73.3) | 8 (42.1) | 0.092 |
| Copy number alteration | |  |  |  |
| *EGFR* amplification/gain | | 9 (60) | 12 (63.2) | 1.0 |
| *PTEN* hemi/homozygous deletion | | 6 (40) | 9 (47.4) | 0.74 |
| *CDKN2A* hemi/homozygous deletion | | 9 (60) | 11 (57.9) | 1.0 |
| *PDGFRA* amplification/gain | | 2 (13.3) | 2 (10.5) | 1.0 |
| *CDK4* amplification | | 2 (13.3) | 5 (26.3) | 0.43 |
| *MDM2* amplification | | 0 (0) | 4 (21.1) | 0.11 |
| *NFKBIA* hemizygous deletion | | 1 (6.7) | 5 (26.3) | 0.20 |
| *TP53* hemizygous deletion | | 2 (13.3) | 5 (26.3) | 0.43 |
| Postoperative treatment | |  |  |  |
| Radiation therapy + temozolomide | | 11 (73.3) | 18 (94.7) | 0.15 |
| None | | 4 (26.7) | 1 (5.3) |  |
| Recurrence pattern | |  |  |  |
|  | Non-local recurrence | 3 (33.3) | 4 (26.7) | 1.0 |
|  | Local recurrence | 6 (66.7) | 11 (73.3) |  |
|  | None | 2 | 4 |  |
|  | Unknown | 4 | 0 |  |

^a^Mann-Whitney U test was applied for age, and Fisher’s exact test was applied for all others.

**Supplementary Table 3 Clinical and molecular features of butterfly glioblastoma in Public-cohort and ALL-cohort based on the subtypes**

|  | Public-cohort | | | ALL-cohort | | |
| --- | --- | --- | --- | --- | --- | --- |
|  | CC-type (n = 12) | Hemispheric-type (n = 34) | P-value^a^ | CC-type (n = 27) | Hemispheric-type (n = 53) | P-value^a^ |
| Age at diagnosis, median (range), years | 59 (23-75) | 59 (31-83) | 0.72 | 59 (23-76) | 59 (31-83) | 0.52 |
| Sex, female | 7 (58.3%) | 11 (32.4%) | 0.17 | 16 (59.3%) | 22 (41.5%) | 0.16 |
| Tumor resection | 12 (100%) | 34 (100%) |  | 19 (70.4%) | 49 (92.5%) | **0.017** |
| Biopsy | 0 | 0 |  | 8 (29.6%) | 4 (7.5%) |  |
| *MGMT*p methylation | 10 (83.3%) | 16 (47.1%) | **0.043** | 22 (81.5%) | 25 (47.2%) | **0.0039** |

^a^Mann-Whitney U test was applied for age, and Fisher’s exact test was applied for all others. P values < 0.05 are in bold.

|  | CC-rate = 30% | | CC-rate = 40% | | CC-rate = 50% | | CC-rate = 60% | | CC-rate = 70% | |
| --- | --- | --- | --- | --- | --- | --- | --- | --- | --- | --- |
| Introduced variables | Hazard ratio (95%CI) | P-value | Hazard ratio (95%CI) | P-value | Hazard ratio (95%CI) | P-value | Hazard ratio (95%CI) | P-value | Hazard ratio (95%CI) | P-value |
| Subtypes |  |  |  |  |  |  |  |  |  |  |
| CC-type | 1.1 (0.71-1.8) | 0.57 | 1.7 (1.0-2.8) | 0.050 | 1.8 (1.1-3.0) | **0.033** | 1.3 (0.78-2.3) | 0.29 | 1.4 (0.79-2.4) | 0.27 |
| Hemispheric-type | Ref. |  | Ref. |  | Ref. |  | Ref. |  | Ref. |  |
| Age^*^ | 1.01^#^ (0.99-1.03) | 0.20 | 1.01^#^ (0.99-1.03) | 0.23 | 1.01^#^ (0.99-1.03) | 0.23 | 1.01^#^ (0.99-1.03) | 0.20 | 1.01^#^ (0.99-1.03) | 0.21 |
| Sex, male | 1.1 (0.71-1.8) | 0.60 | 1.1 (0.70-1.8) | 0.62 | 1.1 (0.69-1.8) | 0.69 | 1.1 (0.69-1.8) | 0.67 | 1.1 (0.71-1.8) | 0.61 |
| *MGMT*p |  |  |  |  |  |  |  |  |  |  |
| Unmethylation | 1.5 (0.93-2.5) | 0.098 | 1.7 (1.0-2.9) | **0.039** | 1.8 (1.1-3.0) | **0.030** | 1.6 (0.98-2.7) | 0.063 | 1.6 (0.97-2.7) | 0.066 |
| Methylation | Ref. |  | Ref. |  | Ref. |  | Ref. |  | Ref. |  |

**Supplementary Table 4 Multivariate analysis of independent prognostic factors associated with overall survival in the ALL-cohort** **based on** **CC-rates of 30%, 40%, 50%, 60%, and 70%**

^*^continuous variables, ^#^ hazard ratio for 1 unit change, P values < 0 .05 are in bold

**Supplementary Table 5** **Multivariate analysis of independent prognostic factors associated with overall survival in the K-cohort and Public-cohort**

|  | K-cohort | | Public-cohort | |
| --- | --- | --- | --- | --- |
| Introduced variables | Hazard ratio (95%CI) | P-value | Hazard ratio (95%CI) | P-value |
| CC-type | 2.4 (1.0-5.6) | 0.052 | 4.3 (1.9-9.8) | **0.0009** |
| Age^*^ | 1.01^#^ (0.97-1.06) | 0.55 | 1.06^#^ (1.03-1.09) | **0.0003** |
| Sex | 0.82 (0.35-2.0) | 0.66 | 0.71 (0.37-1.4) | 0.31 |
| *MGMT*p |  |  |  |  |
| Unmethylation | 1.6 (0.64-4.2) | 0.29 | 4.0 (1.9-8.5) | **0.0003** |
| Methylation | Ref. |  | Ref. |  |

^*^continuous variables, ^#^ hazard ratio for 1 unit change, P values < 0.05 are in bold.

**Supplementary Table 6** **Multivariate analysis of independent prognostic factors associated with overall survival in the ALL-cohort with only tumor resected cases**

| Introduced variables | Hazard ratio (95%CI) | P-value |
| --- | --- | --- |
| CC-rate^*^ | 1.01^#^ (1.0-1.02) | **0.030** |
| Age^*^ | 1.03^#^ (1.0-1.05) | **0.039** |
| Sex | 1.2 (0.73-2.1) | 0.43 |
| *MGMT*p |  |  |
| Unmethylation | 1.6 (0.94-2.8) | 0.081 |
| Methylation | Ref. |  |

^*^continuous variables, ^#^ hazard ratio for 1 unit change, P values < 0.05 are in bold.
